# Preferences for sexual health services among middle-aged and older adults in the UK: a discrete choice experiment

**DOI:** 10.1136/sextrans-2024-056236

**Published:** 2024-09-12

**Authors:** Yoshiko Sakuma, Warittha Tieosapjaroen, Dan Wu, Hayley Conyers, Thomas Shakespeare, John Guigayoma, Fern Terris-Prestholt, Stephen W Pan, Joseph D Tucker, Jason Ong, Eneyi Kpokiri

**Affiliations:** 1 Department of Clinical Research, London School of Hygiene & Tropical Medicine, London, UK; 2 Melbourne Sexual Health Centre, Carlton, Victoria, Australia; 3 Central Clinical School, Monash University, Melbourne, Victoria, Australia; 4 Department of Social Medicine and Health Education, School of Public Health, Nanjing Medical University, Nanjing, China; 5 Department of Population Health, London School of Hygiene & Tropical Medicine, London, UK; 6 Department of Behavioral and Social Sciences, Brown University School of Public Health, Providence, Rhode Island, USA; 7 Department of Global Health and Development, London School of Hygiene & Tropical Medicine Faculty of Public Health and Policy, London, UK; 8 Warwick Medical School, University of Warwick, Coventry, UK; 9 The University of Texas at San Antonio, San Antonio, Texas, USA; 10 Institute for Infectious Diseases and Global Health, The University of North Carolina at Chapel Hill, Chapel Hill, North Carolina, USA

**Keywords:** SEXUAL HEALTH, HEALTH SERVICES RESEARCH, Epidemiology

## Abstract

**Abstract:**

**Objectives:**

Sexual health is an integral part of well-being. However, the sexual health needs and desires of middle-aged and older adults have been largely disregarded. Therefore, this study aimed to understand the sexual health service preferences of adults aged 45 and older to improve the accessibility of sexual health services in the UK.

**Methods:**

The formative stage of the discrete choice experiment (DCE) followed three steps: concept elicitation, refining and implementation. The attributes and levels were determined through 22 semistructured interviews during the concept elicitation, followed by pilot testing for refining the survey. Qualtrics XM, with conjoint project features, was implemented as the DCE survey platform. We used a random parameter logit model to estimate the relative importance (RI) of each attribute and preference for each attribute level. We also used a latent class model to explore groups of participants with similar preferences.

**Results:**

In total, 200 responses were included for analysis. The demographic breakdown included 62.5% females, 35.5% people with disabilities and 26.0% identifying as a sexual minority. The median age was 53. Preferences for using sexual health services were mainly influenced by the mode of delivery (RI 32%), location (RI 18%) and cost (RI 16%). Participants showed a preference for face-to-face interactions at sexual health clinics and displayed a willingness to pay for private services. Extra support and the consultation style played minor roles in their decision-making process. No differences in preferences were identified among disabled people. However, sexual minorities expressed their preferences for conventional messaging.

**Conclusions:**

Our study revealed that middle-aged and older individuals prioritise sexual health services offering face-to-face consultations, emphasising a preference to attend sexual health clinics over cost. Aligning service delivery with these preferences has the potential to significantly improve the accessibility and uptake of sexual health services for adults aged 45 and older in the UK.

**WHAT IS ALREADY KNOWN ON THIS TOPIC**

- Around 80% of middle-aged and older people in the UK are still sexually active, and a study revealed that the number of new HIV diagnoses among middle-aged and older people has doubled over the past decade. However, the sexual health of middle-aged and older adults has long been neglected by various sectors of research and policy. This study, therefore, aimed to explore preferences for sexual health services for middle-aged and older people in the UK and to improve access and uptake.

**WHAT THIS STUDY ADDS**

- The factors influencing the choice of sexual health services among middle-aged and older adults were the mode of delivery and location over cost. This population group showed a preference for face-to-face consultations at sexual health clinics when receiving sexual health services and a willingness to pay for private services. There were no differences in the service preferences of people with disabilities, but sexual minorities showed a preference for conventional messages.

**HOW THIS STUDY MIGHT AFFECT RESEARCH, PRACTICE OR POLICY**

- Middle-aged and older people in the UK prioritised sexual health services that offer face-to-face consultations with inclusive messaging, emphasising a preference to attend sexual health clinics over cost. Aligning service delivery with these preferences has the potential to improve the accessibility and uptake of sexual health services for this population in the UK.

## Introduction

 Sexual health is an integral part of the mental and physical well-being of an adult.^1^ The WHO has acknowledged the need to optimise access to sexual health services for middle-aged and older adults.^2^ However, most sexual health services, social interventions and research have focused on younger people. The sexual health needs and desires of middle-aged and older people have been largely neglected in policy, healthcare practice and even research scope.^3^

Contrary to misperceptions that older individuals are not sexually active,^4^ more than 80% of those aged 50–90 in the UK remain sexually active.^5^ Moreover, the number of new diagnoses of HIV among individuals aged 50 and older in the UK has risen from 9% to 16% over the past decade.^6^ Additionally, it is projected that by 2028, over half of the individuals receiving HIV care will be over the age of 50.^6 7^ Middle-aged and older adults face an increased risk of having sexual health issues due to the intersection of ageing, disability and healthcare access challenges. This combination of factors puts this population at higher risk for sexually transmitted infections and other sexual health difficulties.

In the UK, attendance at sexual health clinics among middle-aged and older people is quite low, with rates below 1.5% in Britain.^8^ Moreover, when older individuals attempt to access sexual health services, their health-seeking journeys for help with sexual health needs often conclude without reaching a satisfactory service or solution.^8 9^ Although there is an obvious need for sexual health services to be inclusive of middle-aged and older people to provide services more closely tailored to their needs, much of the data on preferences related to sexual health services have focused on younger populations.^10 11^ Few studies have examined the preferences of middle-aged and older adults related to sexual health.

This discrete choice experiment (DCE) aimed to identify the sexual health service preferences of adults aged 45 and older in the UK, including disabled people (assessed by the Washington Group tool) and sexual minorities (non-heterosexual).

## Methods

We employed a DCE methodology to explore preferences. A DCE survey is a method that allows individuals’ preferences for healthcare interventions and services to be quantitatively elicited.^12^ We employed the Washington Group tool to assess self-reported functional limitations. In this study, we classified individuals as disabled if they reported ‘a lot of difficulty’ or were ‘unable to do’ any activity in any domain of the tool.^13^ For non-heterosexual participants, we consistently used the term ‘sexual minorities’ throughout the study. The formative stage of the DCE followed three steps (online supplemental file 1).

### Stage 1. Concept elicitation

#### Attributes and levels

In this initial phase, we conducted 22 semistructured interviews from October 2021 to July 2022, followed by performing a thematic analysis, and theory of participation action research to determine the final attributes and levels to be used in the DCE survey. The study primarily used convenience sampling and recruited participants through snowball sampling methods. Purposive sampling was also employed to engage disabled older adults through ‘Independent Living Alternatives’, a social enterprise that works with disabled older adults. The final attributes, levels and definitions of attribute levels are shown in table 1.

**Table 1:** Finalised attributes and levels

Attributes	Levels	
Service type		Definition
Service provider	GP clinic	Typically, your local GP, where you are registered and provide basic and general healthcare services.
	Sexual health clinic	Specialised in the management of sexual health-related conditions and enquiry.
	Online platform	Sexual health services by healthcare professionals entirely online.
Mode of delivery	Face to face	Attend GP clinic or sexual health clinic appointment in person.
	Telephone call	Have a sexual health consultation with doctors/nurses over the phone.
	Videoconference	Have a sexual health consultation with doctors/nurses via online video call.
Cost (out of pocket)	NHS (free)	Getting free sexual health services from the NHS.
	Private (£50–£100)	Paying £50–£100 to receive sexual health services from a private sexual health clinic.
	Private (over £100)	Paying over £100 to receive sexual health services from a private sexual health clinic.
**Providercharacteristics**		
Consultation communication style	Patient centred	Receiving sexual health services in a warm, empathetic, friendly and professional manner.
	Not patient centred	Receiving sexual health services in a strict professional manner.
**Serviceexperience**		
Additional support	Family, friend or personal assistant	Having a family or friend accompany you to attend a sexual health consultation.
	Healthcare professional from the clinic	Having an HCP instead of family or friends accompany you to attend a sexual health consultation.
	No one	Independently attend sexual health services.
Accessibility of facilities, equipment and messaging	Accessible facilities, inclusive equipment	Sexual health services within settings with facilities inclusive of middle-aged and older adults, disabled people and sexual minorities.
	Conventional facilities and equipment	Sexual health service provision in regular settings with generally accepted facilities. Not necessarily tailored to meet the needs of subgroups.
	Accessible messaging	Sexual health services with inclusive language considering different subgroups use a variety of communication tools such as written, verbal, braille and sign language.
	Conventional messaging	Sexual health services are provided in regular settings and use verbal or written communication tool but not tailored to meet the needs of a wide variety of subgroups.

GP, general practitioner; HCP, healthcare professional; NHS, National Health Service.

### Stage 2. Refining

In the second stage, we used convenience sampling methods to recruit 12 participants (including 10 people from a community engagement group composed of local stakeholders, residents, health professionals and local leaders who have an interest in the research topic) for the pilot testing. The pilot test aimed to identify potential issues with the DCE survey in general (eg, cognitive burden, incoherent questions or lack of accessibility). The preliminary results from the pilot test were used for developing full-scale DCE survey.

#### Experimental design

Qualtrics XM was used to present the DCE survey (online supplemental file 2). Participants were each asked to complete six choice sets of two alternatives and an opt-out. We used a D-efficient experimental design to increase the precision of parameter estimates.^14^ We applied constraints to combinations of attributes and levels that could not realistically occur (eg, online platform service provider and face-to-face mode of delivery). The survey consisted of the DCE prompt (included providing definitions and descriptions of the attributes and their levels before participants started answering the DCE surveys, and presenting these in the choice sets), the DCE questions, and questions on sociodemographic information, health conditions and health service utilisation. The survey took approximately 15 min to complete.

The survey was implemented with a text-to-read function that can be accessed by people with visual impairments. Also, the study used pictorial images to represent each attribute/level to ensure that the visual graphics effectively represented our intended meaning. One-to-one support sessions with study staff were also provided via Zoom and telephone to complete the survey if requested.

### Stage 3. Implementation

#### Recruitment strategy

We contacted UK community-based organisations for older adults and sexual health research teams within a UK university to send the online survey link through email. For online recruitment, we advertised the survey through weekly posts on social media (Twitter, Instagram, Facebook, LinkedIn) from April 2023 to October 2023. In addition, we placed flyers (with the survey QR code) at older adult conferences, community centres, local libraries, community cafes and council halls. During the survey period, additional information and support sessions were offered to people who expressed interest in participating in the study.

#### Eligibility criteria

We recruited participants who met all the following criteria: aged 45 years or older, resident in the UK for the last 6 months and willing to provide informed consent. We chose 45 years as our cut-off age as menopause, erectile dysfunction and other sexual health issues become more common after that age.^15^ Participants of the DCE survey were entered into a raffle draw; 20 participants were randomly selected to receive a £50 voucher. Using Orme’s equation, assuming a maximum of four attribute levels, six choice tasks per respondent and two alternatives, the estimated minimum sample size was 167 responses.^12^

### Patient and public involvement

Partnering with citizens, sharing their perspectives and making collaborative decisions, as outlined in Arnstein’s ‘ladder of citizen participation’,^16^ are essential components of genuine public involvement. Throughout the study, eight online meetings were held, engaging a range of public partners, including community engagement groups, community representatives, community-based organisations, researchers and general practitioners (GPs). These stakeholders were integral to the study design, recruitment, dissemination, interpretation of data and reporting of survey results, fostering continuous collaboration and partnership.

### Analysis

#### Statistical analysis

We used a random parameter logit (RPL) model with 1000 Halton draws to estimate the relative importance of each attribute and preference for each attribute level. Relative importance was the percentage of each attribute range divided by the total range of all attributes. All parameters were assumed to have a normal distribution. A positive coefficient indicated a relatively desired attribute level, and a negative coefficient indicated an undesirable level. A statistically significant SD indicated heterogeneity in participants’ preferences for that attribute level. RPL models with interaction terms were used to explore preference heterogeneity among those with different disability statuses (yes/no) and sexual identities (heterosexual/others). The potential uptake of different sexual health service packages (ie, most preferred, status quo, least preferred) was compared using data from the RPL model, which predicted the probability that respondents choose a specific alternative from a set of choices. Additionally, we used a latent class model (LCM) to explore groups of participants sharing similar preferences. In this model, individuals are assigned to classes with specific probabilities. The optimal number of classes was determined based on interpretability, the lowest Akaike information criterion and the log-likelihood function and consultation with experts in DCE and public health. All attribute levels were effects coded. NLOGIT (V.6, Econometric Software, USA) was used for all model estimations.

#### Sensitivity analysis

Sensitivity analyses were also conducted to ensure the reliability of the main analysis by including participants who did not complete the survey but answered at least one DCE choice set.

#### Quality control

To control the quality of the analysis, we sought to identify speeders (defined as those who are more than two SDs from the median duration within the Qualtrics system) or bots (Q_RecaptchaScore is <0.5). Additionally, we introduced captcha authentication (reCAPTCHA technology) questions at the beginning of the survey to verify whether the respondent is a real human being.

## Results

In total, 314 respondents took part in the DCE questionnaire. Of these, 114 respondents did not achieve a 100% completion rate and 200 respondents were included in the final analysis. No responses from speeders or bots were detected in the study. The respondents included 71 (35.5%) disabled people and 52 (26%) sexual minorities. Almost half of the respondents (109, 54.5%) were aged between 45 and 54 years, and only two respondents (1%) were aged 75 or over. In addition, 125 (62.5%) were female, 162 (81%) were white and 75% had at least a college or university degree. Most responses were 187 (93.5%) from England, 8 (4%) from Scotland and 5 (2.5%) from Wales, and 120 (60%) were currently in a relationship, of which 102 (85%) were in a relationship involving sex. The respondents’ sociodemographic characteristics are presented in table 2.

**Table 2:** Demographic characteristics

Demographic categories	FrequencyTotal (%)n=200	People with disability (%)n=71	Sexual minorities (%)n=52
Age (years)			
45–54	109 (54.5)	34 (47.9)	29 (55.8)
55–64	62 (31)	25 (35.2)	17 (32.7)
65–74	20 (10)	7 (9.9)	3 (5.8)
Over 75	2 (1)	2 (2.8)	2 (3.8)
Missing	7 (3.5)	3 (4.2)	1 (1.9)
Sex registered at birth			
Male	73 (36.5)	27 (38.0)	30 (57.7)
Female	125 (62.5)	43 (60.6)	22 (42.3)
Prefer not to say	2 (1)	1 (1.4)	0 (0)
Sexuality			
Heterosexual/straight	148 (74)	46 (64.8)	0 (0)
Gay/lesbian	30 (15)	13 (18.3)	30 (57.7)
Bisexual	10 (5)	6 (8.5)	10 (19.2)
Asexual	3 (1.5)	1 (1.4)	3 (5.8)
Queer	6 (3)	3 (4.2)	6 (11.5)
Pansexual	1 (0.5)	1 (1.4)	1 (1.9)
Other	2 (1)	1 (1.4)	2 (3.8)
Ethnicity			
Asian, Asian British or Asian Welsh	16 (8)	7 (10)	6 (11)
Black, black British, black Welsh, Caribbean or African	20 (10)	5 (7)	2 (4)
Mixed or multiple	0 (0)	0 (0)	0 (0)
White	162 (81)	58 (82)	44 (85)
Other ethnic group	2 (1)	1 (1)	0 (0)
Education			
Primary school	1 (0.5)	0 (0)	1 (1.9)
Secondary school up to 16 years	17 (8.5)	6 (8.5)	4 (7.7)
Higher or secondary or further education	26 (13)	9 (12.7)	1 (1.9)
College or university	77 (38.5)	24 (33.8)	20 (38.5)
Postgraduate degree	73 (36.5)	30 (42.3)	26 (50.0)
Prefer not to say	6 (3)	2 (2.8)	0 (0)
Location			
England	187 (93.5)	68 (95.8)	49 (94.2)
Scotland	8 (4)	0 (0)	1 (1.9)
Wales	5 (2.5)	3 (4.2)	2 (3.8)
Northern Ireland	0 (0)	0 (0)	0 (0)
Marital/relationship status			
Married and living with your spouse	100 (50)	27 (36.6)	15 (28.8)
In a registered same-sex civil partnership	3 (1.5)	1 (1.4)	3 (5.8)
Living with a partner, as a couple	15 (7.5)	4 (5.6)	7 (13.5)
In a steady relationship, but not living together	15 (7.5)	5 (7.0)	5 (9.6)
None of the above	67 (33.5)	35 (49.3)	22 (42.3)
In a relationship			
Yes	120 (60)	34 (47.9)	29 (55.8)
No	80 (40)	37 (52.1)	23 (44.2)
Relationship involving sex (n=120)			
Yes	102 (85)	28 (82.4)	25 (86.2)
No	12 (10)	5 (14.7)	3 (10.3)
Prefer not to say	6 (5)	1 (2.9)	1 (3.4)

**Figure 1:**
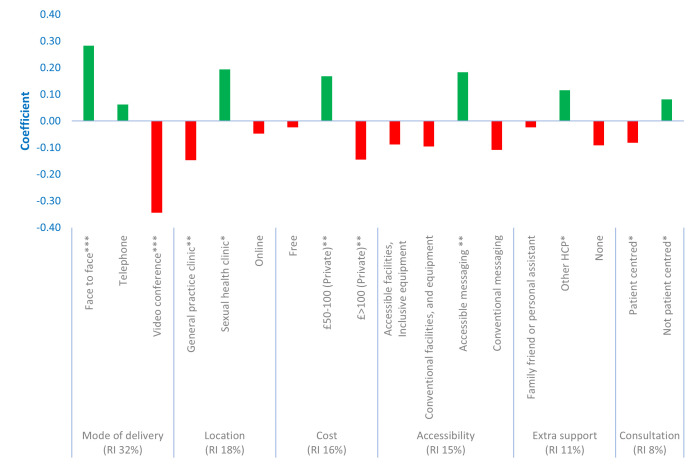
Preferences for sexual health services (n=200). Akaike Infomation Criterion/Number of observations=2.170. Log-likelihood function=−1276.2. ***P<0.01, **p<0.05, *p<0.10. HCP, healthcare professional; RI, relative importance.

The most important factor influencing the utilisation of sexual health services among middle-aged and older individuals was identified as mode of delivery (relative importance 32%), followed by location (relative importance 18%), cost (relative importance 16%), accessibility (relative importance 15%), additional support (relative importance 11%) and consultation (relative importance 8%) (figure 1). The most favoured configuration of sexual health services, yielding an 84% uptake, encompassed face-to-face interactions and consultations conducted in a non-patient-centred manner (eg, in a strict professional manner) at a sexual health clinic. This preferred service is coupled with accessible messaging (eg, inclusive language, variety of communication tools), has a fee range of £50–£100 and is accompanied by a health professional during clinic visits. In comparison, the least preferred programme predicted a 44% uptake. This least preferred programme included patient-centred (eg, warm, empathetic, friendly and professional manner) video consultation with a GP with a fee of more than £100, had no extra support during the visit and accessing only conventional facilities (eg, in regular settings with generally accepted facilities) (onlinesupplemental files 3  4).

Non-heterosexual respondents (52/200) exhibited a preference for conventional messaging (eg, regular setting, use verbal or written communication) and a dislike for accessible messaging when accessing sexual health services, in contrast to their heterosexual counterparts (online supplemental file 5). No significant difference in preferences for sexual health services was found in those with or without a disability (online supplemental file 6).

Four classes were identified in the LCM analysis (table 3), with class 1 (8%), referred to as ‘Uncertain’, comprising participants who randomly selected alternatives. Class 2 (26%), referred to as ‘Reluctantly attend General Practice’, was more likely to opt out of sexual health services. They preferred face-to-face consultations at a GP clinic with family support during sexual health visits. People belonging to this class were more likely to identify as a sexual minority. Class 3 (13%), referred to as ‘Reluctantly attend sexual health clinic’, was less likely to use sexual health services. They preferred free services using inclusive languages at a sexual health clinic, either with healthcare professional support or without support. They disliked online patient-centred services using professional languages with family support and accessing only conventional facilities and equipment. People belonging to this class were more likely to identify as a sexual minority. Class 4 (54%), referred to as ‘Happy to pay’, was more likely to use a sexual health service, with a preference for a fee range of £50–£100 and the use of inclusive language during visits. They disliked accessing sexual health services at a GP clinic with a fee exceeding £100 and a service which only had conventional facilities and equipment.

**Table 3:** Subgroups with similar preferences for sexual health services

	Class 1Uncertain		Class 2Reluctantly attend general practice		Class 3Reluctantly attend sexual health clinics		Class 4Happy to pay	
	Randomly selecting		A sexual minority who tended to opt out preferred face-to-face consultation with family support at a GP clinic.		A sexual minority who tended to opt out preferred free service with accessible messaging at a sexual health clinic with healthcare professional support or no support. They disliked online patient-centred services using professional languages with family support and accessing only conventional facilities and equipment.		This class was more likely to use the service. They preferred a fee of £50–£100 and accessible messaging. They disliked accessing sexual health services at a GP clinic with a fee exceeding £100 and a service which only had conventional facilities and equipment.	
Size	7.7%		25.7%		12.5%		54.1%	
Disable	−1.64678		0.26126		0.48874		Reference class	
Sexual minority	0.94619		0.87578*		1.36587*		Reference class	
	**Coefficient**	**SE**	**Coefficient**	**SE**	**Coefficient**	**SE**	**Coefficient**	**SE**
Location								
GP clinics	−4.72	161.09	0.43**	0.20	0.80	0.49	−0.15*	0.08
Sexual health clinics	24.17	561.76	−0.10	0.21	1.77***	0.58	0.09	0.08
Online	−19.46	425.75	−0.33	0.23	−2.57***	0.88	0.06	0.08
Mode of delivery								
Face to face	17.28	184.18	0.35*	0.20	−0.06	0.54	0.13	0.09
Telephone	54.19	545.75	0.02	0.21	0.24	0.34	−0.05	0.07
Videoconference	−71.46	673.55	−0.38*	0.22	−0.18	0.59	−0.09	0.09
Cost								
Free	−11.35	188.04	−0.17	0.24	0.96**	0.47	0.03	0.08
£50–£100	15.91	165.68	0.05	0.22	−0.11	0.44	0.15**	0.08
>£100	−4.56	136.42	0.12	0.21	−0.84	0.52	−0.18**	0.08
Consultation								
Patient centred	−4.39	90.57	−0.05	0.15	−0.72**	0.32	−0.03	0.05
Not patient centred	4.39	91.57	0.05	0.15	0.72**	0.32	0.03	0.05
Extra support								
Family, friends or personal assistants	10.86	377.67	0.37*	0.22	−3.60**	1.13	−0.01	0.05
Other healthcare professionals	19.74	338.39	−0.23	0.24	1.75***	0.58	0.06	0.07
None	−30.60	642.98	−0.14	0.23	1.86***	0.71	−0.05	0.07
Accessibility								
Accessible facilities, inclusive equipment	−36.32	221.63	0.20	0.28	0.35	0.75	−0.03	0.10
Conventional facilities and equipment	83.95	995.03	−0.11	0.26	−2.14***	0.76	−0.15*	0.09
Accessible messaging	−47.64	430.89	−0.08	0.28	1.79***	0.64	0.18*	0.10
Conventional messaging	−54.39	797.92	0.23	0.26	−1.86***	67	−0.06	0.10
Neither	22.42	353.24	2.11***	0.22	2.96***	0.72	−2.02***	0.20

AIC/NAkaike Infomation Criterion/Number of observations=1.840.

Log -Llikelihood function=−1042.2.

***P<0.01, **p<0.05, *p<0.10.

GP, general practitioner.

### Sensitivity analysis

In our sensitivity analysis, which included individuals who did not answer all the choice sets, the attribute level of patient-centred service p value changed to >0.10, whereas the cost attribute of >£100 p value changed to <0.01 (online supplemental file 7).

## Discussion

We explored the preferences of middle-aged and older adults about using sexual health services in the UK. Few studies have focused on identifying their preferences related to sexual health. Key findings from our study revealed that the majority of them prioritised the mode of delivery and location over the cost of accessing sexual health services, suggesting a willingness to pay for preferred services. Meanwhile, sexual minority groups, who were less likely to access sexual health services, exhibited preferences for specific service configurations. Extra support and consultation style were the least influential factors in their decision-making. This study extends the literature by including adults aged 45 and older, people with disabilities and sexual minorities, making accommodations during implementation and using a DCE.

We found that middle-aged and older people in the UK prefer face-to-face consultations over videoconference consultations. This aligns with another UK study, which showed that the acceptability of video consultation on sexual health was associated with younger age.^17^ This finding may be related to concerns about insufficient online privacy and lower trust and less familiarity in technologies among these population.^18^ Our data suggest that middle-aged and older adults in the UK prefer attending a sexual health clinic rather than a general practice for their sexual health needs. This assertion is supported by international studies, such as those conducted in Norway, indicating that GPs may have less training, support and knowledge in sexual health for this population group compared with specialist physicians.^19^,^21^

Our results indicate that cost is not the most important factor driving access to sexual health services among middle-aged and older adults; they were willing to pay to receive private sexual health services rather than attending National Health Service (NHS) for free services. In the UK, NHS sexual health services have long been challenged by limited capacity and increased waiting times due to the shortage of staff and resources. Currently, only 7% of UK practices can meet the 48-hour appointment target, falling short of government expectations.^22^ The long waiting times and extensive process to access sexual health services, coupled with the current system’s inadequate response to the sexual health needs of middle-aged and older people, are significant factors driving them to ‘reluctantly’ pay for private services. This underscores the need for enhanced service delivery within the NHS to meet both governmental targets and patient needs effectively.

The study also identified a significant gap in sexual health service utilisation among middle-aged and older adults: while the ideal service configuration has an expected uptake of 84%, only 54.1% are classified in a group that are likely to use sexual health services. This discrepancy may stem from older adults often not seeing themselves as typical service users or may not recognise the need for such services, as supported by findings that approximately 80% of them did not seek professional help or advice for their sexual lives.^23^ Regarding access to sexual health services, it is crucial not to overlook their sexual health service needs. A significant portion of middle-aged and older adults exhibit reluctance towards paying high fees for accessing sexual health services. However, for some participants, private sexual health services are so desired that they may be willing to pay for them. Enhancing health professionals’ training and scaling up services for middle-aged and older adults are necessary steps to deliver the ‘specialised’ and ‘inclusive’ services that they desire for their sexual health needs.

This study’s strength is rooted in its quantitative nature to elicit middle-aged and older individuals’ preferences for sexual health services in the UK, a demographic frequently overlooked in sexual health research. By quantifying these preferences, the study offers a deeper understanding of the needs of this age group. However, we noted several limitations. First, our sample is not fully representative of the UK’s middle-aged and older population, as over 90% of the participants were from England, and 81% were white.^24^ Our sample under-represented people from rural areas and people outside of England. Second, the study’s reliance on online methods for participant recruitment and questionnaire responses might have inadvertently excluded a segment of middle-aged and older individuals with limited internet literacy. Notably, a significant portion of the study participants (85.5%) were middle aged (45–64 years), which necessitates caution when extrapolating these findings to those aged 65 and above. The sensitivity of the subject matter and the observed reluctance among them to discuss sexual health issues point towards the potential need for in-person or on-site recruitment strategies in clinical settings. Additionally, our questionnaire does not allow us to identify which specific channel each respondent comes from. We acknowledge this as a limitation of our study and have explicitly addressed it for future research on such topics. Third, although the attributes included in this study were selected through comprehensive interviews with the target population, we acknowledge that there may be other attributes not covered in this study that could also influence the preferences of the participants. Fourth, hypothetical bias is inherent in stated preference surveys.^25^ Some participants may lack the experience or knowledge to fully grasp the attribute levels presented in this survey, potentially leading to inflated stated willingness to use sexual health services. We mitigated this issue by pilot testing the survey to ensure that participants understood the survey. Fifth, while concerns regarding external validity remain common in stated choice surveys, DCEs have demonstrated strong evidence for external validity.^25^,^27^

## Conclusion

Our study revealed that middle-aged and older people in the UK prioritised sexual health services that offer face-to-face consultations with inclusive messaging, emphasising a preference to attend sexual health clinics over cost. Aligning service delivery with these preferences has the potential to significantly improve the accessibility and uptake of sexual health services for them in the UK.

## Supplementary material

10.1136/sextrans-2024-056236 online supplemental file 1

10.1136/sextrans-2024-056236 online supplemental file 2

10.1136/sextrans-2024-056236 online supplemental file 3

10.1136/sextrans-2024-056236 online supplemental file 4

10.1136/sextrans-2024-056236 online supplemental file 5

10.1136/sextrans-2024-056236 online supplemental file 6

10.1136/sextrans-2024-056236 online supplemental file 7

## Data Availability

No data are available.
