# Supplementary material for: Preferences for sexual health services among middle-aged and older adults in the UK: a discrete choice experiment
Source: Sex Transm Infect. 2024 Sep 12;101(3):e056236. doi: 10.1136/sextrans-2024-056236 (PMC12015010; doi:10.1136/sextrans-2024-056236)

Supplementary File 1. Three-stage process for Discrete Choice Experiment (DCE) from concept elicitation to analysis

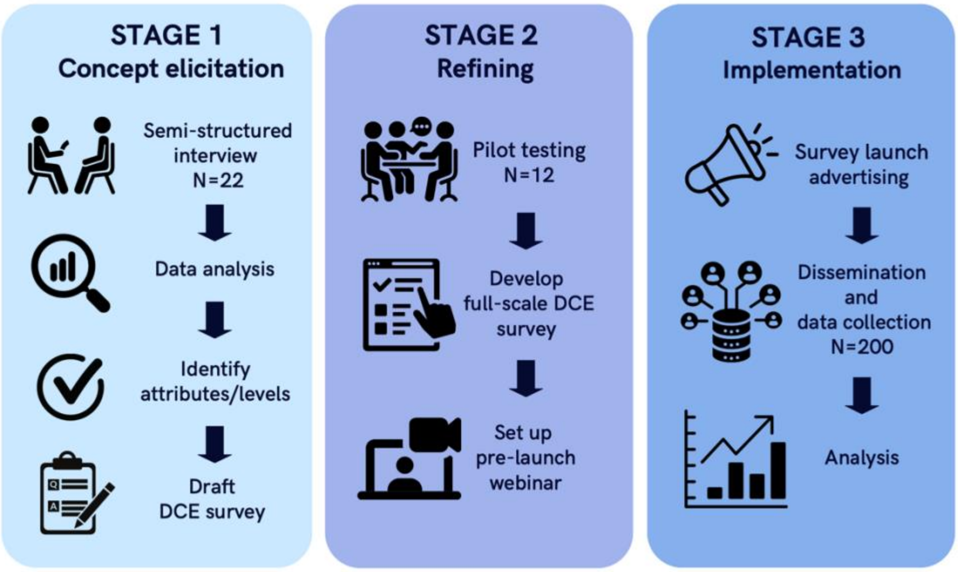

Supplement: online supplemental file 1 [file sextrans-101-3-s001.pdf]
