# Supplementary material for: Preferences for sexual health services among middle-aged and older adults in the UK: a discrete choice experiment
Source: Sex Transm Infect. 2024 Sep 12;101(3):e056236. doi: 10.1136/sextrans-2024-056236 (PMC12015010; doi:10.1136/sextrans-2024-056236)
Supplement: online supplemental file 2 [file sextrans-101-3-s002.pdf]

Supplementary File 2. An example of a choice set. We will give you six choice sets in total (n/6).

	Option 1	Option 2	None
**Provider**	**GP Clinic**<br>Typically, your local GP. where you are registered and provides basic and general health care services.	**Online Platform**<br>Sexual health service by healthcare professionals entirely online.	None of these options
**Mode of delivery**	**Telephone Call**<br>Have a sexual heath consultation with doctors/nurses over the phone.	**Video Conference**<br>Have a sexual health consultation with doctor/nurses via online video call.	
**Cost (out-of pocket)**	**Private (£50-100)**<br>Paying £50-100 to receive sexual health services from a private sexual health clinic.	**NHS (Free)**<br>Getting free sexual health services from the NHS.	
**Consultation communication**	**Patient Centred**<br>Receiving sexual health service in a warm, empathetic, friendly and professional manner.	**Not Patient Centred**<br>Receiving sexual health services in a strict professional manner	
**Additional support**	**No One**<br>Independently attend sexual health services.	**Health Care Professional (HCP) from clinic**<br>Having a HCP instead of family or friends accompany you to attend sexual health consultation.	
**Accessibility of facilities, equipment, and messaging**	**AccessibleMessaging**<br>Sexual health services with inclusive language considering different subgroups, use a variety of communication tools such as written, verbal braille, and sign language.	**Conventional Messaging**<br>Sexual health services are provides in regular settings, use verbal or written communication tool but not tailored to meet the needs of a wide variety of subgroups.	
	○	○	○
