# Supplementary material for: Preferences for sexual health services among middle-aged and older adults in the UK: a discrete choice experiment
Source: Sex Transm Infect. 2024 Sep 12;101(3):e056236. doi: 10.1136/sextrans-2024-056236 (PMC12015010; doi:10.1136/sextrans-2024-056236)
Supplement: online supplemental file 3 [file sextrans-101-3-s003.pdf]

Supplementary File 3. The most vs least preferred service configurations

**Service configurations**	**Uptake**	**Mode of delivery**	**Location**	**Cost**	**Accessibility**	**Extra support**	**Consultation**
**Worst**	44%	Video conference	GP	>£100	Conventional messaging	None	Patient-centred
**Status Quo**	66%	Face-to-face	GP	Free	Conventional messaging	None	Not patient-centred
**Best**	84%	Face-to-face	Sexual health clinic	£50-£100	Accessible messaging	Other healthcare professionals	Not patient-centred
