# Supplementary material for: Preferences for sexual health services among middle-aged and older adults in the UK: a discrete choice experiment
Source: Sex Transm Infect. 2024 Sep 12;101(3):e056236. doi: 10.1136/sextrans-2024-056236 (PMC12015010; doi:10.1136/sextrans-2024-056236)
Supplement: online supplemental file 4 [file sextrans-101-3-s004.pdf]

Supplementary File 4. Preferences for sexual health service (N=200)

Attribute	Relative importance	Attribute level	Coefficient	SE	SD	SE of SD
Mode of delivery	32	Face to face	0.28***	0.08	0.48***	0.16
		Telephone	0.06	0.07	0.00	0.11
		Video conference	-0.34***	0.08	0.48***	0.11
Location	18	General practice clinic	-0.15**	0.07	0.40*	0.23
		Sexual health clinic	0.19*	0.08	0.40***	0.11
		Online	-0.05	0.07	0.01	0.20
Cost	16	Free (NHS)	-0.02	0.07	0.40*	0.22
		£50-£100 (Private)	0.17**	0.07	0.01	0.19
		>£100 (Private)	-0.14**	0.07	0.40***	0.12
Accessibility	15	Accessible facilities, Inclusive equipment	-0.09	0.08	0.02	0.33
		Conventional facilities and equipment	-0.10	0.08	0.01	0.16
		Accessible messaging	0.18**	0.09	0.00	0.16
		Conventional messaging	-0.11	0.09	0.02	0.26
Extra support	11	Family friend or personal assistant	-0.02	0.07	0.00	0.27
		Other healthcare professional	0.12*	0.06	0.00	0.18
		None	-0.09	0.07	0.00	0.21
Consultation	8	Patient-centred	-0.08*	0.05	0.27***	0.09
		Not patient-centred	0.08*	0.05	0.27***	0.09

SD=Standard deviation, SE=Standard error
*** p-value <0.01, ** p-value <0.05, * p-value <0.10
AIC/N = 2.170
Log Likelihood Function=-1276.191
