# Supplementary material for: Preferences for sexual health services among middle-aged and older adults in the UK: a discrete choice experiment
Source: Sex Transm Infect. 2024 Sep 12;101(3):e056236. doi: 10.1136/sextrans-2024-056236 (PMC12015010; doi:10.1136/sextrans-2024-056236)
Supplement: online supplemental file 6 [file sextrans-101-3-s006.pdf]

**Supplementary File 6. Preference heterogeneity for sexual health service between people with and without disability**

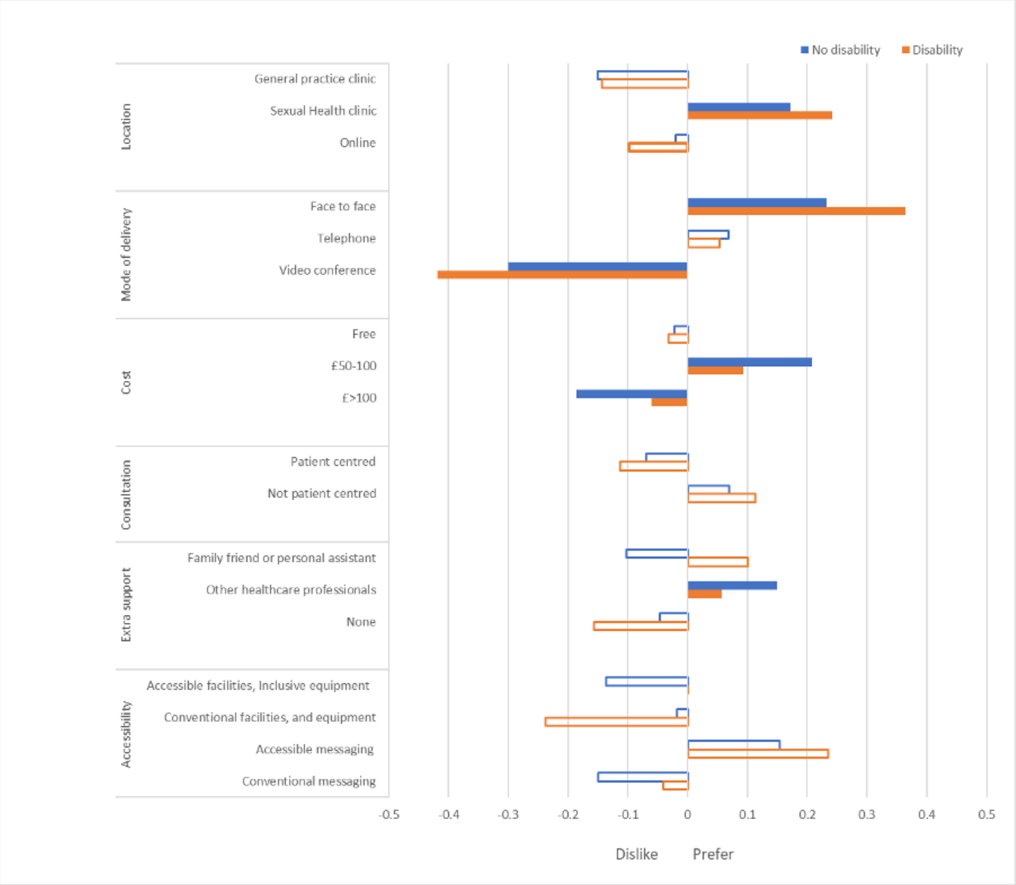


AIC/N = 2.185

Log Likelihood Function=-1273.1

Filled bars indicate a significant preference for an attribute level within each group.

Empty bars indicate an insignificant preference for an attribute level within each group.
