# Supplementary material for: Preferences for sexual health services among middle-aged and older adults in the UK: a discrete choice experiment
Source: Sex Transm Infect. 2024 Sep 12;101(3):e056236. doi: 10.1136/sextrans-2024-056236 (PMC12015010; doi:10.1136/sextrans-2024-056236)
Supplement: online supplemental file 7 [file sextrans-101-3-s007.pdf]

Supplementary File 7. Preference for sexual health service: the results from sensitivity analysis

Attribute	Attribute level	Coefficient	SE	SD	SE of SD
Mode of delivery	Face to face	0.27***	0.07	0.43***	0.15
	Telephone	0.02	0.06	0.00	0.10
	Video conference	-0.29***	0.07	0.43***	0.11
Location	General practice clinic	-0.10*	0.06	0.36*	0.19
	Sexual health clinic	0.19***	0.07	0.36***	0.11
	Online	-0.08	0.07	0.00	0.16
Cost	Free	0.06	0.06	0.38	0.24
	£50-100 (Private)	0.13**	0.06	0.01	0.22
	£>100 (Private)	-0.19***	0.06	0.38***	0.11
Accessibility	Accessible facilities, Inclusive equipment	-0.10	0.08	0.07	0.44
	Conventional facilities and equipment	-0.08	0.07	0.01	0.18
	Accessible messaging	0.18**	0.08	0.00	0.16
	Conventional messaging	-0.10	0.08	0.07	0.38
Extra support	Family friend or personal assistant	-0.06	0.06	0.02	0.36
	Other HCP	0.12**	0.06	0.00	0.17
	None	-0.07	0.06	0.02	0.33
Consultation	Patient-centred	-0.06	0.04	0.25***	0.09
	Not patient centred	0.06	0.06	0.25***	0.09

The sensitivity analysis includes people who only partially completed the DCE survey.
HCP=Health Care Professional

SD=Standard deviation, SE=Standard error

*** p-value <0.01, ** p-value <0.05, * p-value <0.10
